# Signatures of repeated genomic selection associated with human-modified landscapes in genetically independent populations of *Rhinella horribilis*

**DOI:** 10.1038/s41437-026-00831-y

**Published:** 2026-03-12

**Authors:** Gerardo J. Soria-Ortiz, Ella Vázquez-Domínguez

**Affiliations:** 1 Departamento de Ecología de La Biodiversidad, Laboratorio de Genética y Ecología, Instituto de Ecología, Universidad Nacional Autónoma de México, Ciudad de México, Mexico; 2 Posgrado en Ciencias Biológica, Unidad de Posgrado Universidad Nacional Autónoma de México, Ciudad de México, Mexico

**Keywords:** Evolutionary ecology, Molecular evolution

## Abstract

Human-modified environments constitute evolutionary scenarios where novel environmental conditions impose multiple selective pressures on wild species. Rapid adaptation to such environments is critical for species survival. Hence, deciphering the environmental factors associated with species tolerance to modified habitats is fundamental for understanding local adaptation processes across populations. We studied the Giant Toad *Rhinella horribilis* from two landscapes characterized by land-use changes resulting from combined traditional and intensive agriculture and livestock practices. We identified potential outlier loci, assessed genotype-environment associations, annotated candidate genes, and tested for signals of repeated genomic selection in the two landscapes. We used an integrative analytical approach and assessed patterns of genetic repeatability at the genome scale, which improve confidence in identifying true selection signals and provide insights into genetic responses contributing to adaptive evolution. We found positive genotype-environment associations (GEA) related to suboptimal climatic and water physiochemical conditions. Candidate genes were negatively and positively linked with different environmental variables (temperature, solar radiation, oxygen availability, potassium levels in water bodies). Our findings provide evidence of repeated genomic evolution at the functional level, with successful annotation of 34 shared (statistically overlapped) genes between landscapes. Seven genes were enriched for biological processes and metabolic pathways, associated mainly with embryonic development, sexual maturation, and immune responses. These repeated genomic GEA patterns likely reflect rapid local adaptive responses to stressful conditions imposed by these human-modified environments.

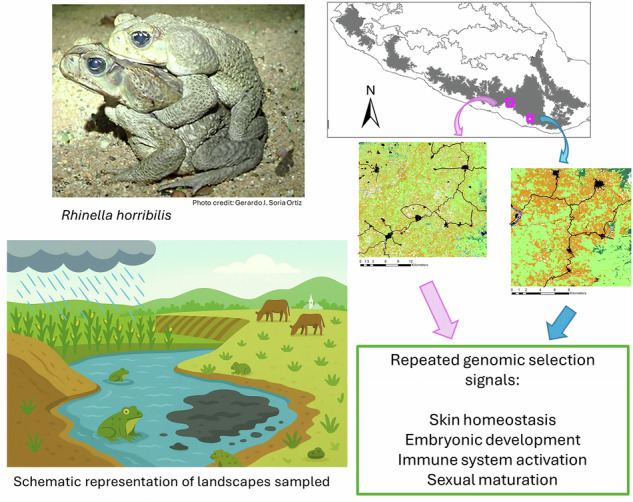

## Introduction

Adaptation can occur within a few generations in response to abrupt environmental changes (Salmón et al. 2021; Garcia-Castillo et al. 2024). One outstanding example of such sudden change is the modification of ecosystems and habitats resulting from human activities. These changes create novel environmental conditions that impose multiple selective pressures on species, making rapid adaptation critical for survival (Johnson and Munshi-South 2017; Exposito-Alonso et al. 2022; Garcia-Castillo et al. 2024; Yves et al. 2025). Human-modified environments constitute evolutionary scenarios characterized by a suite of unique conditions, including land-use change, habitat fragmentation, altered microclimates, and various forms of pollution such as chemicals in soil and water, light, and noise (Bar-Massada et al. 2014; Johnson and Munshi-South 2017; Vázquez-Domínguez et al. 2024). To investigate adaptation under these conditions, genomic analyses of adaptive processes have been conducted in a variety of vertebrate species inhabiting urban landscapes, including fish (*Fundulus heteroclitus*; Osterberg et al. 2018), mammals (*Peromyscus leucopus*; Harris and Munshi-South 2017), birds (*Parus major*; Perrier et al. 2018), reptiles (*Anolis cristatellus*; Winchell et al. 2023), and amphibians (*Rana sylvatica*; Homola et al. 2019). In these examples, signatures of selection appear associated with urbanization, high temperature and salinity levels, metabolic processes, and morphological traits, mainly from standing genetic variation. Such examples highlight the key role of standing genetic variation in facilitating rapid evolution under novel selective pressures (Homola et al. 2019; Exposito-Alonso et al. 2022).

Human-modified environments are expanding globally and pose major threats to wildlife (Salmón et al. 2021). However, some species can persist in these modified landscapes, facilitated by adaptations associated with their life history and functional ecological traits (Bounas et al. 2020; Covarrubias et al. 2021; Soria-Ortiz et al. 2023). Recent evolutionary processes often leave weak signatures of selection in the genome, as allele frequencies rarely reach fixation within a few generations (Salmón et al. 2021). Additionally, human-modified environments can generate false signals of selection in populations due to demographic factors like bottlenecks and genetic drift (Johnson and Munshi-South 2017). To address this, genotype-by-environment association analyses have been effective in detecting genetic variants under weak selection. Also, combining multiple detection methods and overlapping candidate variants can help reduce spurious associations and false positives (Lotterhos et al. 2017; Forester et al. 2018; Ahrens et al. 2021). Regardless of the method used to identify candidate variants, relying on single scenarios can still introduce spurious signals and limit evolutionary inferences (Westram et al. 2021; Babik et al. 2023). Alternatively, evaluating independent replicates provides a more robust framework for identifying consistent and repeated selection signals (Homola et al. 2019; Salmón et al. 2021). This framework helps differentiate between random and neutral processes and those driven by natural selection that shape evolutionary trajectories, thereby providing evidence of parallel evolution (Westram et al. 2021; Cerca 2023; Poore et al. 2023; Semenov et al. 2025).

A complementary approach applied after identifying candidate loci involves examining single or polygenic allele frequency shifts across environmental gradients, annotating variants to genomic regions, and detecting gene expression, target genes, and enriched functions or metabolic pathways, while considering epigenetic modifications or phenotypic traits (Harris and Munshi-South 2017; Homola et al. 2019; Xie et al. 2019; Mueller et al. 2020; Salmón et al. 2021; Caizergues et al. 2022; Babik et al. 2023, 2024; Poore et al. 2023). These integrative strategies enhance our ability to link genotype to phenotype and improve confidence in identifying true selection signals (Xie et al. 2019). Moreover, assessing patterns of genetic repeatability at the genome scale, namely when independently evolving lineages use the same genes to respond to similar selection pressures, provides insights into genetic responses contributing to adaptive evolution (Yeaman et al. 2018; Cerca 2023; Poore et al. 2023).

Amphibians are among the most vulnerable vertebrate groups to land-use change due to their physiological characteristics and life cycle (Cordier et al. 2021; Luedtke et al. 2023). As ectotherms, amphibians’ metabolism and physiology are influenced by ambient temperature, while metamorphosis, which occurs in water bodies, modulates their immune system (Wells 2019; Humphries et al. 2022). These characteristics render them highly sensitive to, among others, microclimatic variation and water pollutants (Cordier et al. 2021; Babik et al. 2024). Despite their vulnerability, amphibians may exhibit rapid adaptation to human-modified environments (Pabijan et al. 2020), which nonetheless remains poorly studied. For example, Homola et al. (2019) detected signatures of selection in genes related to salinity and light pollution in the Wood Frog *Rana sylvatica* from urban versus rural populations, and Babik et al. (2024) reported a decline in genetic diversity at the major histocompatibility complex II (MHC II) with increasing urbanization in smooth newts (*Lissotriton vulgaris*).

The Giant Toad *Rhinella horribilis* is a terrestrial species, with a medium to large body size (100–200 mm total length), that breeds in shallow lotic (stream) and lentic (temporary) water bodies. It is abundant in modified environments (Cortés-Suárez 2017; Soria-Ortiz et al. 2025, 2026) and can tolerate a broad range of climatic variables and water bodies features, conditions that could be considered suboptimal, namely high temperature, evapotranspiration and solar radiation, as well as excess of potassium and sodium ions in the water (De León and Castillo 2015; Soria-Ortiz et al. 2025, 2026). We recently studied *R. horribilis* populations in two landscapes with recent human modification in Oaxaca, Mexico. We demonstrated that these populations are genetically differentiated between landscapes (Soria-Ortiz et al. 2025). Therefore, gene flow between landscapes is not a factor influencing selection signals (Westram et al. 2021), and potential evolutionary processes within each landscape are independent. Furthermore, we also found that environmental variables (solar radiation and relative humidity) significantly influenced *R. horribilis* functional connectivity within landscapes (Soria-Ortiz et al. 2025).

Here, we aimed to evaluate genomic selection signals associated with environmental conditions in these *R. horribilis* populations in the two landscapes (replicates). Our main goals were to (1) identify candidate (outlier) SNPs potentially under selection in two independent landscapes characterized by human-modified environments; (2) assess genotype-environment associations between these loci and climatic and water bodies physicochemical variables; (3) identify candidate genes and determine what biological functions and metabolic pathways are enriched; and (4) test for signals of repeated genomic selection (i.e., similar biological function/metabolic pathway) in the two landscapes. Based on *R. horribilis* life history traits, we predict positive genotype-environment associations reflecting tolerance to suboptimal environmental and water conditions, potentially facilitating local adaptation in these human-modified habitats.

## Materials and methods

### **Sampling and environmental data**

**Fig. 1:**
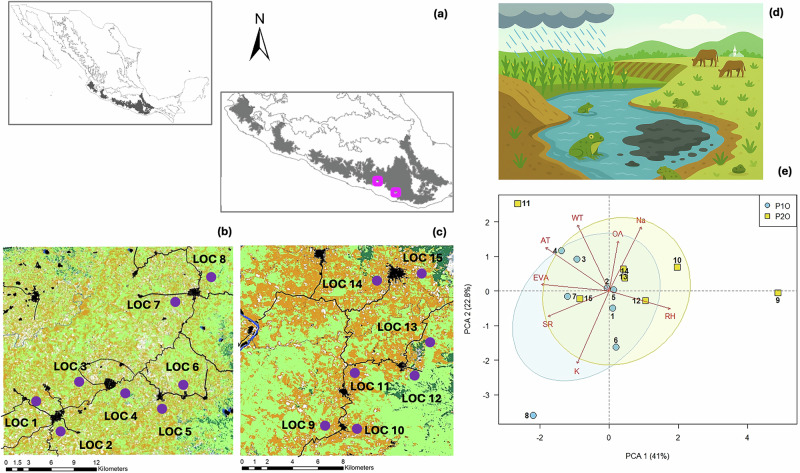
Environmental landscapes within sampling sites per region studied. **a** Sierra Madre del Sur (SMS) at southern Mexico, showing in fuchsia squares. **b** Landscape 1 (P1O) and **c** Landscape 2 (P2O). Within the (**b**, **c**), landscape features are depicted as: urban areas (in black), bare soil (white), agriculture and livestock areas (yellow), secondary forest (light green), dense forest (dark green), water bodies (blue); sampling sites are indicated with purple points. The two landscapes have different scales because they encompass distinct extent of study areas. **d** Schematic representation of the main landscape features where sampling was performed. **e** Principal component analyses (PCA) of the environmental variables evaluated between landscapes (P1O in blue and P2O in yellow). WT water temperature, AT ambient temperature, Na sodium, OA oxygen availability, RH relative humidity, EVA evapotranspiration, SR solar radiation, K potassium.

We worked on two human-modified landscapes in the Sierra Madre del Sur (SMS), Oaxaca, Mexico (P1O and P2O; Fig. 1a–c). Both landscapes are characterized by continuous land-use changes occurring during the last 400 years, mainly as a result of traditional agriculture practices (“the milpa” and “potrero”) and induced pastureland (Pérez-Nicolás et al. 2024). However, approximately 60 years ago this management was intensified across the SMS because of agriculture technification, including monoculture practices and herbicides and insecticides usage (Pérez-Nicolás et al. 2024). Therefore, both landscapes are exposed to similar landscape and water bodies modifications, along with their microclimatic conditions (see Results). Sampling of *R. horribilis* individuals was performed in our previous study Soria-Ortiz et al. (2025). Briefly, we sampled eight sites in P1O and seven in P2O (Fig. 1b, c). Selection of sampling sites was based on the presence of water bodies with aggregation of *R. horribilis* individuals, near urban, agricultural, and livestock areas (Fig. 1d). This sampling scheme allowed us to evaluate physicochemical conditions of water bodies associated with runoff contaminants (e.g., potassium, sodium), as well as climatic variables associated to land-use change. We consider both landscapes as analogous scenarios because the sampling sites share similar selective pressures characterized by high temperature, major incidence of solar radiation, loss of humidity, and aquatic habitats with low oxygen and high temperature and ion concentration. We captured adult individuals randomly at each sampling site during the rainy season (May–September) of 2019. Tissue samples were taken from a toeclip of each individual and stored in 2 ml tubes with 99% ethanol. All individuals were released at their sampling location.

To characterize the local environment of each landscape, we recorded microclimatic and water body variables (Soria-Ortiz et al. 2025, 2026). We used a Kestrel 3000 climate meter (Kestrel® Instruments) to take measures of ambient temperature (AT) and relative humidity (RH). Additionally, we obtained data for solar radiation (SR) and evapotranspiration (EVA) variables from WorldClim 2.1, at a resolution of 1 km, registering the environmental value of the pixel of the sampling sites. We also measured six physicochemical water properties in all the ponds where toads were sampled with a Hanna HI9829 multiparameter meter (HANNA® Instruments): pH (pH), water temperature (WT), oxygen availability (OA), and particles per million (ppm) of potassium (K), nitrates (NO), and sodium (Na).

### DNA extraction, sequencing, and bioinformatics processing

The genomic data used in this study were obtained from one of our previous studies (Soria-Ortiz et al. 2026). Importantly, we here performed completely new filtering and bioinformatics protocols appropriate for GEA and genomic selection analyses (Ahrens et al. 2021; Hemstrom et al. 2024). We extracted genomic DNA from 190*R. horribilis* individuals, 125 and 65 from P1O and P2O, respectively. We use the DNeasy Blood and Tissue Kit (Qiagen, Valencia, CA, USA), following the manufacturer’s protocol. DNA quality and quantity were confirmed on 1% agarose gels using GelRed® (Biotium, Fremont, CA) and a Qubit^TM^ fluorometer, respectively (Invitrogen, Carlsbad, CA). Library preparation and sequencing were performed at the University of Wisconsin Biotechnology Center, following the paired-end ddRAD protocol (Peterson et al. 2012) on an Illumina NovaSeq line to obtain 150 bp paired reads using dual indexing; the restriction enzymes used were *Pstl* and *Bfal* and the size selection range was 500 bp.

We demultiplexed raw reads with the function *process_radtags* in Stacks v.2.62 (Catchen et al. 2013) and quality filtering to remove adapters using FastQC v.0.11.9 (Andrews 2010) with a Phred quality score <20, and trimming reads to length of 100 bp with Trimmomatic v.0.36 (Bolger et al. 2014). We aligned the resulting reads to the *R. marina* reference genome (GenBank assembly: GCA_900303285.1) with the function *BWA mem* in BWA v.0.7.1 (Li and Durbin 2010). We performed calling of single-nucleotide polymorphisms (SNPs) for the total sampling data (190 individuals) with the *ref_map.pl* pipeline and the *populations* module in Stacks, using the parameters *p* = 15, *r* = 0.7.

From this first SNPs dataset we built two datasets, one per landscape (P1O and P2O) and post-quality filtering was subsequently performed for each dataset with VCFtools v.0.1.13 (Danecek et al. 2011), with the following parameters: only biallelic loci were retained; minimum genotype depth (*--minDP*) of 10; minimum genotype quality (*--minGQ*) of 20; maximum amount of missing data of 20% (*--max-missing*); minor allele frequency (*--maf*) of 0.05; loci deviating from Hardy-Weinberg equilibrium (*p* > 0.0001) were removed.

### Identification of potential candidate SNPs and genotype-environment association

We used two approaches to identify potential candidate SNPs in each landscape separately, genotype-environment association (GEA) and outlier (OA) methods. First, to select uncorrelated environmental variables for GEA, we performed Pearson correlation tests (≥0.8) of the climatic and water body physicochemical variables, based on which we removed pH and NO for P1O and pH and AT for P2O. Then we performed a principal component analysis (PCA) with the uncorrelated variables to compare the environmental conditions between the two landscapes.

The first GEA we applied was a redundancy analysis (RDA; Forester et al. 2018) that identifies covarying loci associated with multivariate predictors. We used vegan v.2.6-4 (Oksanen et al. 2012) with the *rda* function in R (R Core Team 2022), with the environmental variables per landscape as explanatory variables and individual genotype data as response variables. We assessed the number of canonical axes with the function *anova.cca* with 1000 permutations, and only significant axes were used to find outlier loci. Loci deviating more than 3 standard deviations from the mean (*p* ≈ 0.001) of each significant axis were retained as candidates (Forester et al. 2018; Capblancq and Forester 2021). Each candidate SNP was assigned to the variable with the highest correlation coefficient (Pearson’s correlation). Secondly, we applied the latent factor mixed model (LFMM; Frichot et al. 2013), which detects correlations between environmental variables and genotypic variation to reveal outliers while controlling for population structure. It is efficient for dealing with false discovery rates, spatially autocorrelated populations (i.e., isolation by distance), and sampling design limitations (de Villemereuil et al. 2014; Lotterhos and Whitlock 2015). We ran LFMM with the function *lfmm_ridge* (Caye et al. 2016) in lfmm v.1.1 available in LEA in R (Frichot and Francois 2015), using individual genotype data as response variables and each environmental variable as explanatory. We ran these analyses with *K* = 3 latent factors for P1O and *K* = 2 for P2O (in accordance with our previous neutral genetic structure results; Soria-Ortiz et al. 2025) and 1000 iterations. We assessed significance levels with the false discovery rate (FDR; Storey and Tibshirani 2003), retaining outlier loci with an FDR *p*-value < 0.01.

We applied an OA method that detects outlier loci based on PCA and assumes that loci excessively related to population structure are candidates for potential adaptation. Additionally, it does not require grouping individuals into populations and can handle admixed individuals. We used the function *pcadapt* in PCAdapt v.4.3.5 in R (Luu et al. 2017). PCAdapt utilizes the genomic inflation factor (GIF) to correct for inflation of the test score at each locus, which can be due to population structure or other confounding factors. We ran this analysis with default parameters, using *K* = 3 and *K* = 2 latent factors for P1O and P2O, respectively; outlier loci with an FDR *p*-value < 0.01 were retained.

Overlap of potential candidate loci identified with different methods enhances confidence in true selection signals while minimizing the likelihood of false positives. However, candidate loci supported by all tested methods can bias the results towards strong selective sweeps, limiting the identification of the least powerful selection candidates (soft sweeps) (Lotterhos and Whitlock 2015; Forester et al. 2018, Ahrens et al. 2021). Therefore, for cross-validating selection and reducing detection of spurious signals, we considered candidate loci the SNPs detected by at least two of the three methods (PCAdapt, RDA, LFMM); these were combined into a single final dataset of candidate SNPs for each landscape.

### Assessment of selection signals

We aimed to identify repeated genomic-selection by evaluating if the candidate SNPs variation responded similarly in the two landscapes to the same environmental variables. To this end, we performed multiple RDAs using different sets of SNPs as response variables and the climatic and physicochemical variables per landscape as explanatory variables (Fig. S1). First, we tested the candidate loci identified uniquely in each landscape (see Results): (a) 609 SNPs in P1O (Set-609-P1O), (b) 237 SNPs in P2O (Set-237-P2O), using their corresponding environmental variables; we also tested (c) the same candidate SNPs of P1O and (d) P2O each in the other landscape variables (Set-609-P2O and Set-237-P1O, respectively). Finally, to compare the overlap of the candidate loci associated with the same climatic and physicochemical variables in the two landscapes with those under random expectations, we used a hypergeometric test (*p* ≤ 0.05) with the *phyper* function in R.

To include neutral loci for comparison in our assessment, we built a neutral SNPs dataset for each landscape by removing the outlier loci identified with the three methods (PCAdapt, RDA, LFMM) from the original SNPs databases. We obtained 6879 and 11,331 putative neutral SNPs for P1O and P2O, respectively. Next, to confirm that the genetic-environmental associations did not occur by chance, we performed RDAs with the neutral datasets (Fig. S1). That is, we tested the neutral SNPs of each landscape (e) SetN-6879-P1O and (f) SetN-11,331-P2O with their own environmental variables. To additionally test for an effect of the candidate loci and of the sample size (number of SNPs), we randomly selected 609 and 237 SNPs from the neutral datasets for each landscape (g) SetRN-609-P1O, (h) SetRN-237-P1O, (i) SetRN-609-P2O and (j) SetRN-237-P2O, assessing the random-neutral loci on their own and on the other landscape environmental variables as well (k, l, m, n; see Fig. S1; Table S1). Hence, we carried out a total of 14 tests, eight evaluating each landscape’s SNPs set with its landscape variables, and six crossed tests, assessing the role of SNPs identified in one landscape in explaining environmental variation in the other landscape (Table S1).

### SNP annotation and enrichment analyses

We identified the contigs that contained the candidate SNPs and annotated them through searches in the *Rhinella marina* (NCBI Taxonomy ID: 8386) and *Xenopus tropicalis* (NCBI Taxonomy ID: 8364) reference genomes, using the BLASTn tool in the NCBI nucleotide database (National Center for Biotechnology Information, https://blast.ncbi.nlm.nih.gov/Blast.cgi; accessed on January 2024) and the Xenbase (https://www.xenbase.org/xenbase/; accessed on January 2024). Only those sequences with an E-value cut-off ≤0.05 and similarity > 80% for a gene were selected as candidate genes. We associated the resultant gene names with the Xenbase_ID identifier using DAVID at NCBI (https://david.ncifcrf.gov; accessed on January 2024).

To test for signs of repeated genomic selection (i.e., similar biological function/metabolic pathway) between landscapes, we first performed gene enrichment using those genes shared (identified independently) by the two landscapes (*N* = 34; see “Results”), with the ShinyGo v.8.0 platform (http://bioinformatics.sdstate.edu/go//; Ge et al. 2020) and *X. tropicalis* as reference, to identify overrepresented GO ontology terms (Ashburner et al. 2000) and KEGG pathways (Kanehisa et al. 2017).

Next, we used the *C*-score index proposed by Yeaman et al. (2018) to estimate how much of repeated adaptation in pairwise contrasts (overlap between landscapes) occurred under a null expectation. This index is based on the probability of observing a given amount of repeatability by chance under a given null hypothesis; it uses the hypergeometric distribution. We applied the *C*_*hyper*_ formula (Yeaman et al. 2018) considering the identified genes enriched for a biological process and KEGG pathway:

$$Ch{yper}=\frac{\left({a}_{s}-\frac{{a}_{x}* {a}_{y}}{{g}_{0}}\right)}{\frac{\sqrt{\left({ax}* {ay}\right)\left({ax}-g0\right)\left({ay}-g0\right)}}{g0-1}}$$

where *g*_*0*_ is the total number of genes included in the test (number of identified genes that are not repeated between landscapes; see Results); *a*_*x*_ and *a*_*y*_ are the number of loci that contribute to selection in each landscape (candidate genes that are shared between landscapes); and *a*_*s*_ is the observed number of overlapping genes that contribute to selection in both landscapes (candidate genes enriched for a biological function and KEGG pathway that overlap between landscapes). The hypergeometric test evaluates if the overlap (the size effect) is greater than expected by chance.

Finally, we determined the relationship and direction between the environment and the allele frequencies of the SNPs linked to genes enriched for GO terms and KEGG pathways. To this end, we performed an RDA using the alternative allele frequency at site level in each landscape of the SNPs associated with the 34 shared candidate genes as response variable, and key landscape environmental variables as explanatory variables. The most important environmental variable for the allele frequencies were selected based on the highest Pearson correlation coefficient.

#### Research Ethics statement

Fieldwork, sampling, and animal care were performed strictly following the guidelines for working with amphibians and reptiles (Beaupre et al. 2004), approved by and following the guidelines of the Ethics Committee Facultad de Ciencias-UNAM (project PN 2271). The scientific collecting permit was obtained from Secretaría del Medio Ambiente y Recursos Naturales (SEMARNAT; 09/K4-1472/09/18).

## Results

We sampled 125 *R. horribilis* adults in P1O and 65 in P2O (Table S2). SNP calling against the reference genome yielded 1,134,057 polymorphic sites. After applying post-quality filters, the datasets comprised 8753 SNPs for P1O and 13,544 SNPs for P2O (24× coverage), which were subsequently used for outlier detection.

### Outlier identification and genotype-environment association

The multivariate PCA of uncorrelated environmental features between landscapes showed no differences regarding climatic and physicochemical variables (Fig. 1e), which suggests high environmental similarity between them. Results of outlier detection per landscape varied with the method used: PCAdapt identified 109 and 226 outlier SNPs in P1O and P2O, respectively; RDA 608 and 212 SNPs, and LFMM 2389 and 2301 (Table S3).

**Fig. 2:**
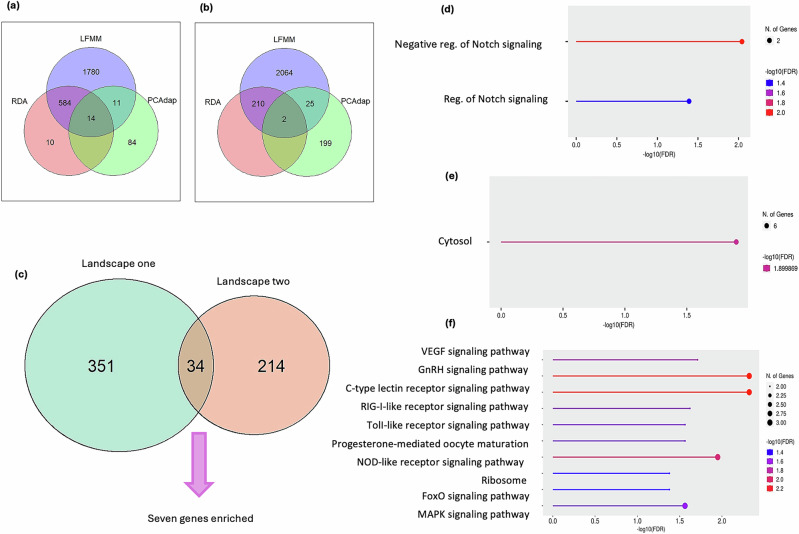
Venn diagrams of the overlap among outlier SNPs. SNPs identified by linear factor mixed models (LFMM), redundant analyses (RDA) and PCAdapt for (**a**) landscape 1 (P1O) and (**b**) landscape 2 (P1O). **c** Venn diagram of the overlap between genes identified in P1O and P2O. Results of the Gene enrichment analysis of the 34 genes shared between the two landscapes are shown for (**d**) GO-biological processes, (**e**) GO-cellular components, and (**f**) KEGG metabolic pathways, depicting the fold enrichment from highest (top) to lowest (bottom). In the latter, only the 10 enrichment pathways with the highest −log10(FDR) are included.

Association results with the climatic and water physicochemical variables showed that LFMM identified associations with the eight variables analyzed in each landscape (Table S3); EVA and WT showed the highest number of associated SNPs in P1O, while in P2O were SR and OA. In turn, RDA identified SNPs positively associated with K and WT in P1O, while negatively with EVA. Associations identified in P2O were all positive, where RH and SR showed a higher number of associated loci (Table S3). The number of loci detected by at least two methods resulted in a total of 609 and 237 candidate SNPs for P1O and P2O, respectively (Fig. 2a, b), which were used for annotation and subsequent analyses.

### Signals of repeated selection

Results of RDA to identify repeated genomic-selection for the different sets of SNPs support that the identified candidate SNPs best explain the landscape’s environmental variation (for detailed results, see Table S1, a-d tests). The candidate SNPs per landscape (Set-609-P1O and Set-237-P2O) explained between 58% and 85% of the environmental variation of their corresponding landscape, respectively (*p* = 0.001), whereby the crossed tests (Set-609-P2O, Set-237-P1O) respectively accounted for 35% and 51% of the variation with the other landscape’s SNPs. Furthermore, these results were supported by the number of candidate SNPs associated with the same climatic and physicochemical variables in each landscape (hypergeometric test, *p* < 0.05; Table S4), indicating that *R. horribilis* responds similarly (not randomly) to independent environmental selection pressures in both landscapes. Most candidate SNPs in P1O were associated with water physicochemical variables (60% individually and 38.9% overlap; Table S4), while in P2O they were associated with climatic variables (78% individually, 42% overlap). When using the neutral (excluding SNPs outliers) and the random SNPs datasets, we could explain very low percentages of the environmental variance of both landscapes, indicating that the detected associations are not spurious (Table S1, e-n tests).

### Functional annotation and genetic-environment associations

The functional annotation of the genomic regions that contained a candidate SNP identified 385 regions associated with at least one gene in P1O and 248 in P2O (*E*-val ≤0.05; Table S5). These genes were considered as the total genes (*g*_*0*_) for the *C*-score index test (Yeaman et al. 2018). Thirty-four genes were shared between landscapes (Fig. 2c), which were considered as those that contribute to selection in each landscape (*a*_*x*_ and *a*_*y*_). Enrichment analyses of these 34 genes assigned seven genes to three main GO terms (GO:0045746, GO:0008593, GO:0005829) and to 13 KEGG metabolic pathways (Table 1 and Fig. 2d–f), which were considered as the overlapped genes that contribute to selection in both landscapes (*a*_*s*_).

**Table 1:** Gene enrichment results of 34 shared genes in the two landscapes (P1O, P2O).

	FDR *p*	N SNPs	Path genes	Fold enrichment	Pathway	Genes
Biological process	0.0090	2	6	237.6559	GO:0045746 negative regulation of NOTCH signaling pathway	TCIM NEUROD1
	0.0407	2	x	83.8786	GO:0008593 regulation of NOTCH signaling pathway	TCIM NEUROD1
Cellular component	0.0125	6	630	6.7902	GO:0005829 cytosol	TCIM EAF2 RPS18 RPS28P9 RABIF TRAF2
Metabolic pathway	0.0050	3	94	21.3752	Path:xtr04625 C-type lectin receptor signaling pathway	MAPK13 PIK3R1 PRKCD
	0.0057	3	85	23.6385	Path:xtr04912 GnRH signaling pathway	MAPK13 MAP3K4 PRKCD
	0.0135	3	145	13.8571	Path:xtr04621 NOD-like receptor signaling pathway	MAPK13 TRAF2 PRKCD
	0.0220	2	53	25.2739	Path:xtr04370 VEGF signaling pathway	MAPK13 PIK3R1
	0.0270	2	66	20.2957	Path:xtr04622 RIG-I-like receptor signaling pathway	MAPK13 TRAF2
	0.0309	3	272	7.3870	Path:xtr04010 MAPK signaling pathway	MAPK13 TRAF2 MAP3K4
	0.0309	2	80	16.7439	Path:xtr04620 Toll-like receptor signaling pathway	MAPK13 PIK3R1
	0.0309	2	90	14.8835	Path:xtr04914 Progesterone-mediated oocyte maturation	MAPK13 PIK3R1
	0.0471	2	125	10.7161	Path:xtr03010 Ribosome	RPS18 RPS28P9
	0.0471	2	135	9.9223	Path:xtr04068 FoxO signaling pathway	MAPK13 PIK3R1
	0.0471	2	144	9.3022	Path:xtr04140 Autophagy-animal	PIK3R1 PRKCD
	0.0471	2	143	9.3672	Path:xtr04210 Apoptosis	PIK3R1 TRAF2
	0.0471	2	145	9.2380	Path:xtr04218 Cellular senescence	MAPK13 PIK3R1

Seven genes were enriched for GO terms (GO; biological process and cellular component) and KEGG metabolic pathways. Only the 16 associations that were significant (FDR *p* ≤ 0.05) are listed. Number of SNPs (N SNPs).

The size effect of the functional overlap calculated with the *C*-score was twice the null expectation and significant (*C*_*hyper*_ = 3.866, expected = 1.93, *SD* = 1.311, *p* = 0.0001; hypergeometric test), indicating an effect of functional processes that contribute to selection in both landscapes (unlikely a random result).

The biological process with the highest significance was the NOTCH signaling (Fig. 2d and Table 2), with two SNPs in each landscape assigned to enriched genes in this system (NEUROD1 and TCIM); they were associated positively with K, SR, and RH, and negatively with AT (Tables 2 and S6). Six SNPs were assigned to cytosol cellular component (Fig. 2e and Table 2). Finally, the most important KEGG metabolic pathways were the C-type lectin receptors (CLR), the NOD-like receptor signaling pathway, the VEGF and MAPK signaling pathways, and the hypothalamic gonadotropin-releasing hormone (GnRH) (Fig. 2f and Table 2). Four and five SNPs for P1O and P2O, respectively, were associated with enriched genes of these metabolic pathways, positively with SR, OA, WT, and K, and negatively with AT and EVA (Tables 2 and S6).

**Table 2:** Genotype-environment associations (GEA) of candidate SNPs associated with seven genes enriched to GO terms and KEGG pathways for *Rhinella horribilis* in two landscapes.

SNP ID	Gen	Environmental predictor	Correlation	Effect	Term/Pathway
P1O					
10330_89	TCIM	Potassium	0.663	+	Notch
34934_85	MAPK13	Potassium	0.736	+	VEGF, MAPK, C-type lectin receptor, NOD-like receptor, RIG-I-like receptor, Toll-like receptor
37417_115	TRAF2	Water temperature	0.451	+	MAPK, RIG-I-like receptor
	PRKCD				C-type lectin receptor, NOD-like receptor
63686_91	MAP3K4	Ambient temperature	0.439	–	MAPK, NOD-like receptor
112556_67	PIK3R1	Water temperature	0.484	+	VEGF, C-type lectin receptor, Toll-like receptor
624633_21	NEUROD1	Ambient temperature	0.403	–	Notch
P2O					
34934_22	MAPK13	Evapotranspiration	0.732	–	VEGF, MAPK, C-type lectin receptor, NOD-like receptor, RIG-I-like receptor, Toll-like receptor
142202_127	NEUROD1	Solar radiation	0.552	+	Notch
290084_106	MAP3K4	Solar radiation	0.533	+	MAPK, NOD-like receptor
444251_58	PIK3R1	Oxygen availability	0.832	–	VEGF, C-type lectin receptor, Toll-like receptor
721346_46	TCIM	Relative humidity	0.746	+	Notch
786000_75	TRAF2	Solar radiation	0.546	+	MAPK, RIG-I-like receptor
813406_90	PRKCD	Solar radiation	0.532	+	C-type lectin receptor

P1O landscape 1, P2O landscape 2. Positive (+) and negative (−) relationships are indicated.

## Discussion

Our findings highlight two important aspects of amphibian evolution in human-modified environments. We demonstrate repeated genomic selection signals in genetically independent populations of the Giant Toad *Rhinella horribilis*. We identified as well positive GEA related to suboptimal climatic and water physiochemical conditions, with genes associated with key genomic functions. These repeated genomic GEA patterns likely reflect rapid local adaptive responses to stressful conditions imposed by human-modified environments.

### Candidate loci and signatures of repeated genomic selection

Given that outlier detection is prone to detect false positive signals we followed protocols that improve detection (Lotterhos et al. 2017; Forester et al. 2018; Ahrens et al. 2021). We applied conservative filters and considered overlapping results from two of three methods applied, which have different outlier detection assumptions. The latter can represent a trade off because reducing the identification of false positives comes at the expense of losing some true positives (Ahrens et al. 2021). Nonetheless, our *R. horribilis* populations are most likely under recent evolutionary selective pressures due to contemporary human activities; thus, we were able to identify soft rather than strong selective sweeps, since adaptive variants are unlikely to become fixed over short time periods (Homola et al. 2019; Salmón et al. 2021). Additionally, by only considering candidate SNPs those overlapping with at least two methods, we increased the probability of identifying true positives (Lotterhos and Whitlock 2015; Forester et al. 2018, Ahrens et al. 2021).

We acknowledge that our sampling size could compromise the detection of true positive variants. However, as Selmoni et al. (2020) indicate, species with limited dispersal —like *R. horribilis*—can yield strong GEA with moderate sample sizes (~200 individuals; five to ten sampling locations). Additionally, the methods used (LFMM, RDA and PCAdapt) are able to detect weak selection signals in scenarios with small sampling sizes, low *F*_*ST*_ values and under neutral scenarios (e.g., isolation by distance) (de Villemereuil et al. 2014; Lotterhos and Whitlock 2015; Forester et al. 2018; Ahrens et al. 2021).

We found that association between candidate SNPs and environmental variables from both landscapes were not random (*p* < 0.001; hypergeometric test). Cross-validation tests showed that candidate SNPs in P2O are mainly associated with climatic variables, whereas those in P1O relate to water physicochemical characteristics. Suboptimal conditions of water bodies in P1O are probably related to runoff from agricultural and urban areas, which alter physicochemical conditions (e.g., Brady et al. 2012, 2013). Comparatively, GEA patterns in P2O may be driven by microclimate changes linked to low vegetation cover and proximity to urban areas. Thus, although both landscapes may exert similar selection pressures, particular landscape modification features influence the number of and the environment-candidate SNPs associations.

Our findings provide evidence of repeated genomic selection at the functional level, with successful annotation of 34 shared genes between landscapes. Seven of these genes were enriched for biological functions and KEGG pathways, and the size effect of the functional overlap was significant (*C*_*hyper*_; Yeaman et al. 2018). This evidence indicates that populations of the two landscapes share embryonic development and immune response processes, which likely contribute to adaptation in these human-modified environments. In this context, the standing genetic variation present within populations is expected to be a greater contributor to rapid adaptation in comparison to novel alleles introduced via mutation or gene flow (Barrett and Schluter 2008). For example, Homola et al. (2019) identified 37 outlier loci for *Rana sylvatica* under a replicated rural/urban environment framework, three of which aligned to scaffolds with annotations associated with proteins. Keller et al. (2023) reported repeated signals of local adaptation in montane populations of the beetle *Chrysomela aeneicollis* driven by variation in snowfall. They found 3–6 SNPs associated with genes coding for proteins involved in intracellular signaling and energetics. Increasing the number of replicates could improve the robustness of our findings, while contrasting them with populations with less or null anthropogenic selection pressures could help distinguish adaptive from neutral patterns.

### Genotype-environment associations and genomic function for adaptive responses

Our findings support our prediction of positive GEA reflecting *R. horribilis* tolerance to suboptimal environmental and water conditions, potentially facilitating local adaptation in these human-modified habitats (Yves et al. 2025). The seven enriched genes that overlap between landscapes were mostly involved with the regulation of the NOTCH signaling that controls multiple mechanisms of cellular differentiation processes during embryonic development in the nervous system and muscular cells (Bray 2016; Favarolo and López 2018; Zhou et al. 2022). The enriched genes were also involved with various KEGG metabolic pathways (MAPK, VEGF). MAPK plays an important role in cellular proliferation, differentiation, development, and apoptosis, and is activated by growth and stress factors (Krens et al. 2006; Wen et al. 2022). In turn, VEGF modulates angiogenesis and organogenesis during embryonic development (Haigh 2008; Vieira et al. 2010; Shibuya 2013). A decreased expression or function loss of the MAPK families (MEK5-ERK5 and p38MAPK) inhibits neuronal differentiation and myogenesis in *Xenopus* embryos and tadpoles (Krens et al. 2006). Notably, while anoxia is associated with an increase in NOTCH receptors and ligands in *R. sylvatica* (Gupta and Storey 2022), hypoxia can upregulate VEGF, stimulate vein growth and increase the VEGF mRNA average life (Haigh 2008). Hence, some environmental conditions can regulate these signals under stressful conditions.

Function and mechanisms of such processes and pathways are relatively well known but the environmental factors that activate and regulate them remain unclear. We detected some environmental variables potentially involved in local adaptation processes (Yves et al. 2025). Candidate loci related to genes pik3r1 and mapk13 (associated with VEGF) were negatively and positively linked with OA and high K levels in water bodies, respectively, while a positive association was found between TCIM, NEUROD1, TRAF2, and MAP3K4 with high WT and K levels (Table 2). These environmental factors are known to affect amphibian development and metamorphosis (Brans et al. 2018; Wells 2019; Covarrubias et al. 2021): low oxygen affect survival and development of tadpoles (Wells 2019), high temperatures accelerate metamorphosis but reduce the sizes of adults (e.g., *Epidalea calamita*, Sanuy et al. 2008; *Bufo gargarizans*, Chen et al. 2021), and high potassium and sodium negatively affect hatching, muscle development and survival in embryos and tadpoles (Wells 2019; Lorrain‑Soligon et al. 2024). In human-modified landscapes, small or temporary water bodies formed at the beginning of the rainy season commonly have high temperatures, high levels of pollutants (e.g., pesticides, sodium, potassium), and shorter hydroperiods with hypoxic conditions (Jiménez and Sommer 2017; Preuss et al. 2020; Goessens et al. 2022). *Rhinella horribilis* is an explosive breeder that frequently uses such temporary water bodies, which impose selective pressures for its development. Thereby, upregulated NOTCH and MAPK could be crucial for the rapid development of embryos, tissue formation, and maintenance of stable osmotic conditions, while VEGF could enhance gas exchange and organ development during metamorphosis. Similar enriched VEGF and MAPK pathways have been reported in the fish *Leuciscus waleckii* inhabiting alkaline and hypoxic water bodies (Wang et al. 2021). Although the reproductive success of *R. horribilis* in these conditions suggests local adaptation, further controlled studies are needed to confirm it.

We also found a positive association between candidate loci enriched for the NEUROD1 gene (present in NOTCH) and SR. Solar radiation has strong impacts on skin homeostasis, which is modulated also by NOTCH signaling promoting keratinocyte differentiation, darker skin pigmentation and tissue repair in response to UV radiation (Mandinova et al. 2008; Zhang et al. 2024). Amphibians have behavioral, physiological and molecular mechanisms for UV radiation protection like skin pigmentation as adaptation strategies (Blaustein and Belden 2003; Kosch et al. 2024). For example, the Cuyaba Dwarf Frog, *Physalaemus nattereri*, increases skin melanocytes after exposure to UV radiation (Franco-Belussi and De Oliveira 2016); similarly, darker phenotypes of the Plateau Brown Frog, *Rana kukunoris*, have shown upregulated genes associated with biosynthesis of melanosomes (Zhang et al. 2024). Hence, the NOTCH signaling could play a protective role for the keratinocytes enriched with melanosomes (Bray 2016; Zhou et al. 2022; Kosch et al. 2024), potentially enabling *R. horribilis* to tolerate higher solar radiation exposure in human-modified environments.

Notably, we also found enrichment of metabolic pathways associated with Pattern Recognition Receptors (PRRs) involved in the innate immune system, including the NOD-like, the C-type lectin and Toll-like receptors that specialize in recognition of fungal β-glucans (Geijtenbeek and Gringhuis 2009; Garcia-Vidal and Carratalà 2012), as well as the RIG-I-like for viral RNA recognition (Rehwinkel and Gack 2020; Jiang et al. 2021). The PPRs recognize infectious agents and activate immune, microbicidal, pro-inflammatory and antiviral responses (Geijtenbeek and Gringhuis, 2009; Grogan et al. 2018; Nie et al. 2018; Varga et al. 2019; Rehwinkel and Gack, 2020; Chuphal et al. 2022). Amphibian’s immune response is influenced by environmental stressors (Yang et al. 2016), and different genes have been associated with immune responses exposed to bacteria, virus and pathogens, like the fungus *Batrachochytrium dendrobatidis* (Bd; Rödin-Mörch et al. 2021; McDonald et al. 2023) or in the invasive populations of *R. marina* in Australia (Brown et al. 2015; Rollins et al. 2015).

Moreover, we found candidate loci related to PRR genes (TRAF2, PIK3R1, MAPK13 and PRCD) associated with water bodies that exhibited high WT and alkaline and anoxic conditions. In water bodies with detrimental conditions pathogens proliferate, modifying the amphibian’s microbiome and increasing the risk of infection (Jiménez and Sommer 2017; Preuss et al. 2020; Goessens et al. 2022; Zhou et al. 2023). Although PRRs have been little studied in amphibians, early activation and regulation for pathogen detection is essential to control infections across life stages. For instance, the RIG-I gene (present in the RIG-I-like receptor pathway) is upregulated in kidney and spleen tissues infected with iridovirus in the Chinese Giant Salamander *Andrias davidianus* (Meng et al. 2020), and Toll-like receptors are associated with antiviral and antibacterial immunity in the Dybovsky’s Frog *Rana dybowskii* (Nie et al. 2018). Similarly, the American Bullfrog (*Rana [Lithobates] catesbeianus*) exhibits upregulation of Toll-like and NOD-like receptor genes in response to *Elizabethkingia miricola* infection (Li et al. 2023). While direct evidence of PRRs in Bd response is incipient, their role in the detection of pathogen antigens is key for activation of the adaptative immune system (Grogan et al. 2018). For example, the toad *Brachycephalus pitanga* presents upregulated genes associated with T-cell receptors in early stages of Bd infection (McDonald et al. 2023). The interaction between the innate and adaptative immune systems is likely related to a rapid and effective response when exposed a second time to the pathogen, suggesting that PRRs can be subject of natural selection against infections (Grogan et al. 2018). These mechanisms can help *R. horribilis* persist in water bodies with suboptimal conditions immersed in human-modified environments and outcompete other local amphibians.

## Conclusions and future directions

Human-modified environments are challenging for amphibian populations, and identifying cases of local adaptation can illuminate how populations deal with those stressful conditions. Our results highlight the importance of evaluating repeated signals of selection to elucidate the role of environmental conditions as mechanisms shaping potential adaptive patterns in response to stressful conditions. The identified genes and metabolic pathways and their associated functions can prove crucial for amphibian adaptation to human-modified environments, allowing individuals to tolerate adverse climatic and water conditions, as suggested by our results. Therefore, our findings provide key hypotheses for future research. For instance, common garden experiments could be designed to assess the interplay of these genes and metabolic pathways in controlled environmental settings to evaluate their effects on morphological characteristics like tadpoles’ survival. It would be interesting to assess functional validation by analyzing the expression of upregulated genes associated with these pathways and the molecular mechanisms acting on tadpole development and survival in mesocosm experiments. While we found signals of repeated genomic evolution in *R. horribilis* in the two landscapes, expanding the study by exploring a larger sampling cover would aid assessing if the potential adaptation variants are present and evaluate their adaptive potential. Finally, comparative studies on syntropic species should be conducted to determine if they respond similarly at the genomic level to analogous selection pressures and to better understand convergence selection.

## Supplementary information

Supplementary Material

Supplementary Table S5

## Data Availability

The demultiplexed Illumina reads of the Giant Toad *Rhinella horribilis* were uploaded to NCBI SRA database under accession number PRJNA1335177 (Soria-Ortiz et al. 2025). Sampling locations, data per individual, methods for all analyses, and genetic results are available in the main text and in the Supporting Information.
