# Supplementary material for: Signatures of repeated genomic selection associated with human-modified landscapes in genetically independent populations of Rhinella horribilis
Source: Heredity (Edinb). 2026 Mar 12;135(4):289–98. doi: 10.1038/s41437-026-00831-y (PMC13102914; doi:10.1038/s41437-026-00831-y)
Supplement: Supplementary file 1 — Supplementary Material [file 41437_2026_831_MOESM1_ESM.pdf]

Signatures of repeated genomic selection associated with human-modified landscapes in genetically independent populations of *Rhinella horribilis*

Supplementary material

Table of Contents

**Figure S1**	
Redundancy analysis (RDA) testing scheme using different sets of SNPs	Page 2
**Tables S1-S6**	
Results of redundancy analysis tests with the different sets of SNPs	Page 3
Sampling sites and individuals	Page 4
Candidate SNPs identified for *Rhinella horribilis* with three methods (RDA, LFMM and PCAdapt)	Page 5
Hypergeometric test results of the overlap of candidate SNPs associated with the same environmental variables in P1O and P2O	Page 6
Gene annotation of candidate SNPs in P1O and P2O	Page 6
Redundancy analysis (RDA) for the association between 34 shared genes and environmental variables in P1O and P2O	Page 7

Figures

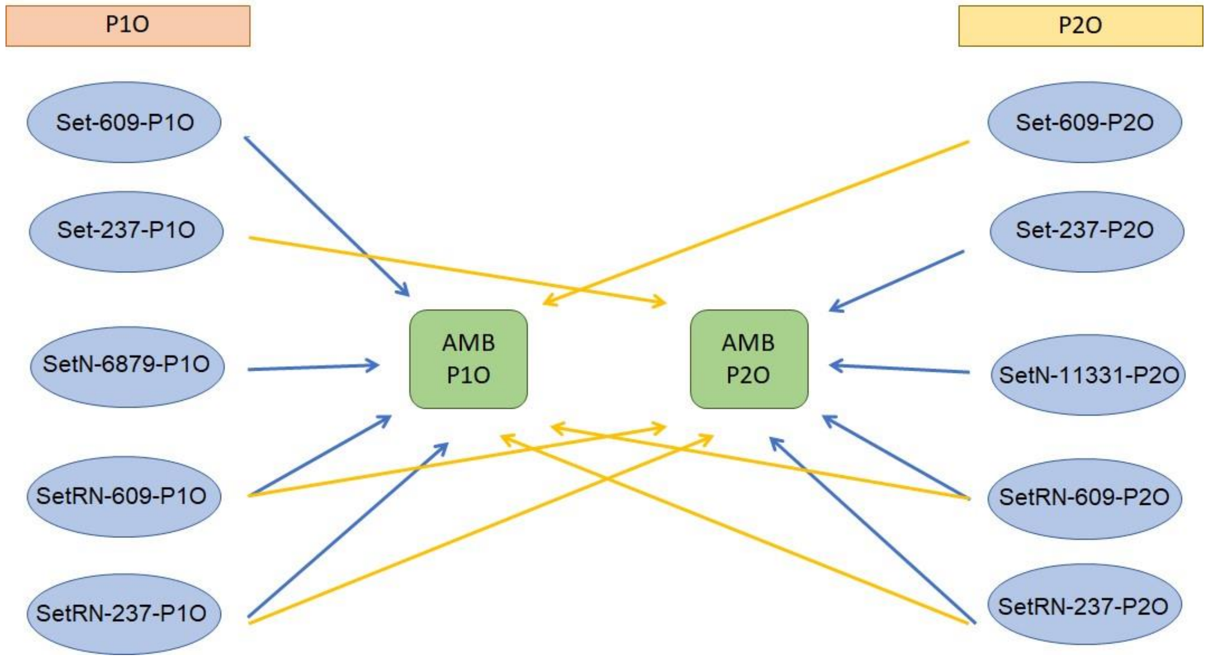

Figure S1. Redundancy analysis (RDA) scheme to test repeated signals of genomic selection and to identify if potentially adaptive genetic variation in the two landscapes responded similarly to the same landscape variables in *Rhinella horribilis* in two landscapes (P1O and P2O). On the left and right sides, the sets of SNPs obtained for P1O and P2O, respectively. The environmental variables of each landscape are schematized in the center (AMB; in green). Blue arrows indicate RDA tests to measure genetic x environmental relationships within the same landscape; yellow arrows indicate RDA tests to evaluate genetic x environmental relationships between landscapes (cross tests). See details in section Assessment of selection signals of the main text.

Tables

Table S1. Results of redundancy analysis (RDA) tests with different sets of SNPs to explain environmental variation within and between the two landscapes. P10: landscape 1, P2O: landscape 2; Amb1: environmental variables of landscape 1; Amb2: environmental variables of landscape 2.

**SNPs sets tested**	R^2 **(%)**	***p***
(a) Set-609-P1O: 609 candidate SNPs P1O – Amb1	58.3	0.001
(b) Set-237-P2O: 237 candidate SNPs P2O – Amb2	85.7	0.001
(c) Set-609-P2O: 609 candidate SNPs P1O – Amb2	35.5	0.001
(d) Set-237-P1O: 237 candidate SNPs P2O – Amb1	51.8	0.001
(e) SetN-6879-P1O: neutral SNPs P1O – Amb1	6.3	0.001
(f) SetN-3494-P2O: neutral SNPs P2O – Amb2	19.4	0.001
(g) SetRN-609-P1O: 609 random neutral SNPs P1O – Amb1	6.7	0.001
(h) SetRN-237-P1O: 237 random neutral SNPs P1O – Amb1	7.1	0.001
(i) SetRN-609-P2O: 609 random neutral SNPs P2O – Amb2	26.0	0.001
(j) SetRN-237-P2O: 237 random neutral SNPs P2O – Amb2	24.4	0.001
(k) 609-P2O-P1O: 609 random neutral SNPs P2O – Amb1	14.0	0.001
(l) 237-P2O-P1O: 237 random neutral SNPs P2O – Amb1	14.6	0.001
(m) 609-P1O-P2O: 609 random neutral SNPs P1O – Amb2	26.1	0.001
(n) 237-P1O-P2O: 237 random neutral SNPs P1O – Amb2	27.2	0.001

Table S2. Sampling within each landscape. Eight sampling sites in landscape 1 (P1O) and seven in landscape 2 (P2O). n = number of individuals.

OP1	Sampling sites	n	Total
	Loc1	10	
	Loc2	15	
	Loc3	11	
	Loc4	23	
	Loc5	23	
	Loc6	10	
	Loc7	23	
	Loc8	10	
			125
**OP2**			
	Loc9	8	
	Loc10	4	
	Loc11	11	
	Loc12	3	
	Loc13	8	
	Loc14	10	
	Loc15	21	
			65

Table S3. Outlier loci detected for *Rhinella horribilis* by redundancy analysis (RDA), latent factor mixed model (LFMM) and PCAdapt methods. Number of SNPs associated with each variable (total for PCAdapt) and its percentage are indicated for landscape 1 (P1O) and landscape 2 (P2O).

**P1O**		**Method**			
**Environmental variable**	**RDA**	**LFMM**	**PCAdapt**	**% RDA**	**% LFMM**
Ambient temperature (AT)	56	412		8.5	10.6
Solar radiation (SR)	17	282		2.8	7.3
Relative humidity (RH)	0	201		0	5.2
Evapotranspiration (EVA)	141	1235		23.2	31.9
Potassium (K)	196	197		32.2	5.1
Sodium (Na)	27	551		4.4	14.2
Oxygen availability (OA)	0	215		0	5.6
Water temperature (WT)	171	772		28.1	19.9
Total (unique loci)	608	2389	109	100	100
Total (with duplicated loci)		3865			100
**P2O**		**Method**			
**Environmental variable**	**RDA**	**LFMM**	**PCAdapt**	**%RDA**	**%LFMM**
Solar radiation (SR)	45	1946		21.2	31.5
Relative humidity (RH)	107	936		50.5	15.2
Evapotranspiration (EVA)	21	755		9.9	12.2
Potassium (K)	0	1		0	0.01
Sodium (Na)	0	322		0	5.2
Nitrates (NO)	0	107		0	1.7
Oxygen availability (OA)	25	1081		11.8	15.5
Water temperature (WT)	14	1020		6.6	16.5
Total (unique loci)	212	2301	226	100	100
Total (with duplicated loci)		6168			100

Table S4. Hypergeometric test of the overlap of candidate SNPs associated with the same environmental variables in each landscape. P1O: landscape 1; P2O: landscape 2. CLI: climatic variables (evapotranspiration, solar radiation, ambient temperature, relative humidity); PHS: physicochemical variables (oxygen availability, water temperature, sodium, potassium). Between parentheses is the percentage of candidate SNPs in relation to the total in each case.

**609 candidate SNPs identified in P1O**				
	P1O-609	P2O-609	Overlap	*p* (hypergeometric test)
CLI	253 (41.5)	227 (37.2)	98 (16.1)	**< 0.001**
PHS	365 (59.9)	395 (64.9)	237 (38.9)	**< 0.001**
**237 candidate SNPs identified in P2O**				
	P1O-237	P2O-237	overlap	*p* (hypergeometric test)
CLI	184 (77.6)	138 (58.2)	112 (42.2)	**< 0.001**
PHS	43 (18.1)	87 (36.7)	17 (7.2)	**< 0.001**

Table S5. Gene annotation table for 385 regions associated with at least one gene in landscape 1 (P1O) and 248 in landscape 2 (P2O); 34 genes found in both landscapes are in bold (**available as an independent excel file**).

Table S6. Redundancy analysis (RDA) for the association between the 34 shared genes and environmental (AT, SR, RH, EVA) and water physicochemical (K, NA, OA, WT) variables in landscape 1 (P1O) and landscape 2 (P2O). The best predictor (BP), correlation value (Cor), and gene symbol (Gen S) are indicated. The enriched genes are shown in bold.

					Environmental predictor							
**P1O**												
**SNP ID**	**RDA loading**	**AT**	**SR**	**RH**	**EVA**	**K**	**NA**	**OA**	**WT**	**BP**	**Cor**	**Gen S**
5940_5	0.310	0.135	-0.144	-0.070	0.175	-0.024	0.440	-0.260	0.527	WT	0.527	U2AF2
5941_108	0.190	0.121	-0.150	-0.040	0.169	-0.026	0.433	-0.236	0.473	WT	0.473	ZCCHC3
												SP3
												ANKRD45
												ZFR
												NFYC
**10330_89**	0.407	-0.030	0.550	-0.499	0.014	0.663	-0.554	-0.428	-0.401	**K**	**0.663**	**TCIM**
29533_38	-1.219	-0.529	0.135	0.200	-0.162	0.258	0.155	-0.071	-0.208	AT	0.529	TGFBRAP1
												EMC8
												HELZ
												CBX6
**34934_85**	0.334	-0.178	0.521	-0.393	-0.062	0.736	-0.533	-0.583	-0.431	**K**	**0.736**	**MAPK13**
**37417_115**	0.214	0.103	-0.036	-0.119	0.197	0.058	0.401	-0.332	0.451	**WT**	**0.451**	**STK39**
												ZNF451
												**TRAF2**
												**PRKCD**
												SPNS1
												RABIF
												FRZB2
43017_23	0.427	-0.011	0.550	-0.558	0.040	0.710	-0.485	-0.487	-0.319	K	0.710	USP45
43325_96	-0.120	-0.055	0.383	0.210	0.370	-0.206	0.065	-0.078	-0.040	SR	0.383	RPS28P9
43325_95	-0.125	-0.097	0.446	0.125	0.320	-0.202	0.064	-0.044	0.002	SR	0.446	RPS18

43325_93	0.002	-0.038	0.408	0.138	0.337	-0.238	0.041	-0.055	0.000	SR	0.408	
43326_87	0.203	-0.193	0.006	-0.195	-0.524	-0.172	-0.213	0.012	-0.063	EVA	0.524	
43326_60	0.148	-0.181	-0.073	-0.218	-0.569	-0.215	-0.135	0.084	0.022	EVA	0.569	
57470_27	-1.239	-0.455	0.161	0.124	-0.127	0.247	0.136	0.018	-0.215	AT	0.455	LOC100497432
											SLC7A9
**63686_91**	-1.087	-0.439	0.075	0.158	-0.191	0.189	0.100	0.041	-0.207	**AT**	**0.439**	**PECAM1**
											**MAP3K4**
**112556_67**	0.176	0.042	-0.173	0.002	0.084	-0.071	0.444	-0.257	0.484	**WT**	**0.484**	**NAB2**
											**PIK3R1**
114880_71	0.403	-0.048	0.499	-0.451	0.011	0.701	-0.531	-0.483	-0.398	K	0.701	SOX11
156350_66	-1.190	-0.496	0.031	0.246	-0.178	0.151	0.211	0.074	-0.121	AT	0.496	SLAMF8
											MANSC1
623828_63	-1.162	-0.439	0.130	0.153	-0.131	0.210	0.123	0.044	-0.205	AT	0.439	TNIK
**624633_21**	-1.040	-0.403	0.044	0.157	-0.148	0.145	0.205	-0.017	-0.098	**AT**	**0.403**	**NEUROD1**
879041_124	0.516	-0.055	0.493	-0.376	0.015	0.655	-0.591	-0.458	-0.434	K	0.655	KMT2C
**P2O**												
**34934_22**	-0.109	-0.589	0.339	-0.481	-0.732	-0.225	0.234	0.301	-0.395	**EVA**	**0.732**	**MAPK13**
72018_43	-0.139	-0.182	0.271	0.220	-0.051	-0.253	-0.323	0.228	-0.459	WT	0.459	U2AF2
92393_74	-0.297	0.535	-0.617	0.751	0.231	-0.121	0.427	0.654	0.488	RH	0.751	FRZB2
126617_49	1.082	-0.385	0.535	-0.073	-0.449	-0.261	0.232	-0.245	0.346	SR	0.535	RPS28P9
											RPS18
**142202_127**	1.092	-0.434	0.552	-0.074	-0.451	-0.338	0.294	-0.270	0.356	**SR**	**0.552**	**NEUROD1**
**290084_106**	1.086	-0.378	0.533	-0.051	-0.464	-0.357	0.322	-0.221	0.304	**SR**	**0.533**	**MAP3K4**
444250_65	-0.437	0.218	-0.427	0.466	-0.165	-0.295	0.560	0.832	0.136	OA	0.832	NAB2
**444251_58**	-0.437	0.218	-0.427	0.466	-0.165	-0.295	0.560	0.832	0.136	**OA**	**0.832**	**PIK3R1**
582279_123	-0.467	0.235	-0.472	0.469	-0.142	-0.239	0.552	0.836	0.205	OA	0.836	ZCCHC3
689120_38	1.085	-0.390	0.537	-0.079	-0.458	-0.267	0.242	-0.244	0.345	SR	0.537	PECAM1
698786_122	-0.194	-0.381	0.224	-0.320	-0.577	-0.283	0.254	0.354	-0.484	EVA	0.577	BLTP3B
721346_50	-0.302	0.544	-0.627	0.746	0.237	-0.119	0.437	0.648	0.490	RH	0.746	ZNF451
**721346_46**	-0.302	0.544	-0.627	0.746	0.237	-0.119	0.437	0.648	0.490	**RH**	**0.746**	**TCIM**
722973_16	1.078	-0.324	0.528	-0.044	-0.498	-0.350	0.316	-0.157	0.223	SR	0.528	
750597_88	-0.272	0.563	-0.645	0.802	0.230	-0.163	0.498	0.688	0.531	RH	0.802	KMT2C

750598_12	-0.272	0.563	-0.645	0.802	0.230	-0.163	0.498	0.688	0.531	RH	0.802	
766025_53	1.007	-0.285	0.481	-0.009	-0.452	-0.299	0.266	-0.127	0.240	SR	0.481	SP3
786001_57	1.080	-0.398	0.537	-0.077	-0.472	-0.277	0.256	-0.227	0.347	SR	0.537	ANKRD45
**786000_75**	1.081	-0.398	0.546	-0.067	-0.499	-0.358	0.325	-0.197	0.289	**SR**	**0.546**	**ZFR**
												NFYC
												TRAF2
**813406_90**	1.086	-0.384	0.532	-0.062	-0.435	-0.281	0.248	-0.256	0.352	**SR**	**0.532**	**TNIK**
												**PRKCD**
												SPNS1
												RABIF
827236_55	0.886	-0.561	0.571	-0.155	-0.814	-0.428	0.447	0.137	0.145	EVA	0.814	SLAMF8
												MANSC1
869295_44	-0.251	0.499	-0.604	0.743	0.242	-0.189	0.503	0.595	0.527	RH	0.743	USP45
												LOC100497432
876016_76	1.097	-0.427	0.544	-0.067	-0.437	-0.356	0.318	-0.283	0.365	SR	0.544	SOX11
876016_46	1.085	-0.380	0.524	-0.061	-0.435	-0.272	0.250	-0.252	0.367	SR	0.524	
891384_74	1.150	-0.415	0.571	-0.052	-0.484	-0.332	0.286	-0.239	0.357	SR	0.571	STK39
952796_10	1.074	-0.335	0.515	-0.062	-0.436	-0.179	0.159	-0.225	0.351	SR	0.515	TGFBRAP1
												CBX6
												SLC7A9
												EMC8
												HELZ
